# Measurement of sustainment of prevention programs and initiatives: the sustainment measurement system scale

**DOI:** 10.1186/s13012-020-01030-x

**Published:** 2020-09-03

**Authors:** Lawrence A. Palinkas, Chih-Ping Chou, Suzanne E. Spear, Sapna J. Mendon, Juan Villamar, C. Hendricks Brown

**Affiliations:** 1 Suzanne Dworak-Peck School of Social Work, University of Southern California, Los Angeles, CA USA; 2 Department of Preventive Medicine, Keck School of Medicine, University of Southern California, Los Angeles, CA USA; 3 Department of Health Sciences, California State University, Northridge, CA USA; 4 Center for Prevention Implementation Methodology (Ce-PIM) for Drug Abuse and HIV, Department of Psychiatry and Behavioral Sciences, Northwestern University Feinberg School of Medicine, Chicago, IL USA

**Keywords:** Sustainability, Sustainment, Measurement, Behavioral health, Prevention

## Abstract

**Background:**

Enhancing the sustainability of evidence-based prevention programs for mental and behavioral health requires tools for measuring both sustainability determinants and sustainment outcomes. The aim of this study was to develop the Sustainment Measurement System Scale (SMSS) and to assess its reliability and construct validity for measuring both determinants and outcomes of efforts to sustain prevention programs and initiatives.

**Methods:**

A 42-item scale comprised of items identified from qualitative data collected from 45 representatives of 10 programs and 8 SAMHSA program officers was administered to 186 representatives of 145 programs funded by 7 SAMHSA prevention grant initiatives. Cronbach’s alphas were used to determine inter-item reliability. Convergent validity was assessed by comparisons of a global measure of sustainment with current SAMHSA-funding status and continued operation in the same form. Discriminant validity was assessed by comparisons of sustainability determinants with whether or not the program had undergone adaptations.

**Results:**

Confirmatory factor analysis provided support for a 35-item model fit to the data. Cronbach’s alpha was .84 for the sustainment outcome construct and ranged from .70 to .93 for the sustainability determinant constructs. All of the determinant constructs were significantly associated with sustainment outcome individual and global measures for the entire sample (*p* < 0.01 to 0.001) and for community-based programs and programs with a substance abuse focus (*p* < 0.05 to 0.001). Convergent validity was supported by significant associations between the global sustainment measure and current SAMHSA funding status and continued operation in the same form (*p* < 0.001). Four of the sustainability determinant constructs (responsive to community needs; coalitions, partnerships, and networks; organizational staff capability; and evaluation, feedback, and program outcomes) were also significantly associated with current SAMHSA funding status (*p* < 0.5 to 0.01). With the exception of organizational staff capability, all sustainability determinants were unrelated to program adaptation as predicted.

**Conclusions:**

The SMSS demonstrated good reliability and convergent and discriminant validity in assessing likelihood of sustainment of SAMHSA funded prevention programs and initiatives. The measure demonstrates potential in identifying predictors of program sustainment and as a tool for enhancing the likelihood of successful sustainment through ongoing evaluation and feedback.

Contributions to the Literature

- Understanding of evidence-based practice sustainment requires improved tools for measurement of determinants and outcomes.
- The Sustainment Measurement System Scale (SMSS) is the first tool of its kind that is designed to measure both sustainment determinants and outcomes.
- The SMSS demonstrates good reliability and convergent and discriminant validity.
- The SMSS can be used to conduct audits and provide feedback to grantees committed to sustaining prevention programs and initiatives once initial funding has ended.

## Background

Sustainment is considered to be the final stage of the process of implementation of evidence-based practices, policies, and programs (EBPs) [1, 2]. Defined as “the continued use of program components and activities for the continued achievement of desirable program and population outcomes,” [3] sustainment is considered to have occurred when, after a defined period of time, a program, clinical intervention, and/or implementation strategies continue to be delivered and/or individual behavior change (i.e., clinician, patient) is maintained, either as originally planned or with some degree of adaptation, while continuing to produce benefits for individuals/systems [4]. However, what is to be sustained differs from one program to the next [5, 6]. For instance, with respect to the community coalitions supporting drug and suicide prevention activities, some definitions of sustainment focus on the continued existence of the coalition itself while others focus on the activities and impacts of the coalition [7]. Moreover, sustainability, defined as the capacity to maintain EBP components [8], is increasingly being viewed as a dynamic process with shifting outcomes that represents increased likelihood that the program or infrastructure will continue, while sustainment refers to the continued operation of a program or initiative, often relating to the time after initial funding has ended [8, 9].

In recent years, there has been a proliferation of frameworks that focus specifically on sustainability [3, 4, 7–9]. However, despite the growing consensus as to how sustainability should be defined [3, 4, 9], the underdeveloped state of measurement of sustainment poses one of the most serious methodological challenges to understanding and facilitating sustainability of evidence-based practices and programs [9–11]. Some instruments like the Stages of Implementation Completion (SIC) [12] have been developed to measure the implementation outcomes of a specific intervention [13]. The SIC is an 8-stage assessment tool developed as part of a large-scale randomized implementation trial that contrasted two methods of implementing an EBP for youth with serious behavioral problems in the juvenile justice and child welfare systems. The eight stages range from Engagement (stage 1) with the developers/purveyors in the implementation process, to achievement of Competency in program delivery (stage 8) that span three phases of implementation including pre-implementation, implementation, and sustainability. The SIC was developed to measure a community or organization’s progress and milestones toward successful implementation of Treatment Foster Care Oregon (TFCO) regardless of the implementation strategy utilized. Within each of the eight stages, subactivities are operationalized and completion of activities are monitored, along with the length of time taken to complete these activities. Competency in program delivery (stage 8) is considered to be a measure of program sustainment [13], although continued use of the EBP once competency has been attained is not assessed. Data on the validity and reliability of the SIC to assess sustainment outcomes is lacking. Other instruments like the Program Sustainability Assessment Tool (PSAT) [14–16], Sustained Implementation Support Scale (SISS) [17], and Program Sustainability Index (PSI) [18], take a broader ecological approach to sustainability and are used primarily to assess capacity and to plan for sustainability by measuring determinants or factors that influence sustainability. The PSAT is a 40-item scale, containing 8 sustainability domains (Environmental Support, Funding Stability, Partnerships, Organizational Capacity, Program Evaluation, Program Adaptation, Communications, and Strategic Planning), with 5 items per domain [15], and designed to measure capacity for sustainability a public health program’s capacity for sustainability. The SISS is a 28-item scale composed of five subscales (Program Benefits, Program Burdens, Workplace Support, Workplace Cohesion, and Leadership Style) and designed to measure capacity to maintain implementation of components of an EBP for parent training. The PSI is a 53-item scale reflecting 7 sustainability elements (leadership competence, effective collaboration, understanding the community, demonstrating program results, strategic funding, staff involvement and integration, and program responsivity). All three instruments have demonstrated validity and reliability as measures of a program’s capacity for sustainability. However, none of these instruments assess both sustainability determinants and sustainment outcomes.

One of the reasons for developing a valid and reliable measure of sustainment is to provide a means of conducting an audit and providing feedback to organizations engaged in implementing innovative and evidence-based programs and practices. While monitoring and feedback are recognized as important for prevention [19, 20], much of the relevant science on feedback in health has involved improvement in clinical performance [21–27]. This includes clinical supervision and use of technology like electronic dashboards in measurement-based quality improvement (MBQI) strategies that monitor patient behavior and clinician activity [28–30], while prevention has a more limited history of using computational technologies for monitoring [31–34]. Such feedback offers the clinician a better understanding of whether they are on course to achieve a successful outcome or need to alter their treatment in order to improve the likelihood of a successful outcome. MBQI strategies also hold great promise for facilitating implementation of evidence-based practices [35]. Audit and feedback has demonstrated that it can be an effective strategy for implementation [36, 37], but there is little evidence to suggest that it is equally effective in achieving the sustainment stage of implementation [38].

Federal agencies responsible for wide-scale delivery of prevention programs, including the Substance Abuse and Mental Health Services Administration (SAMHSA), routinely collect information from their grantees to monitor progress toward completion of goals and objectives. SAMHSA supports a wide array of prevention grant programs targeting mental, emotional, and behavioral disorders including illicit substance use, suicide, and antisocial behavior. Each of SAMHSA’s prevention initiatives have specific sets of goals and objectives, and each have different prevention approaches that the administration expects will be sustained once support from SAMHSA is no longer available. As part of their initial proposal for funding, all SAMHSA grantees are required to submit a plan for sustainment of the grantee’s activities once the federal funding has come to an end. SAMHSA programs currently rely on electronic data collection systems including the Transformation Accountability (TRAC) data collection system for SAMHSA’s Center for Mental Health Services (CMHS) programs, and the Coalition Online Management and Evaluation Tool (COMET) and the Performance Management Reporting Tool (PMRT) used by SAMHSA’s Center for Substance Abuse Prevention (CSAP). This information is used to provide feedback to grantees when there is evidence of failure to achieve goals and objectives. However, there is no empirical evidence that such feedback leads to an improvement in performance or increases the likelihood of sustainment.

While SAMHSA remains deeply concerned about sustainment, its mission does not allow monitoring of its grantees after funding has ended. In building a partnership with SAMHSA, the National Institute on Drug Abuse (NIDA)-funded Center for Prevention Implementation Methodology (Ce-PIM, P30DA027828) responded to this need by undertaking the development of a sustainability measure across diverse prevention programs and test its predictive validity over time [39]. This measure was intended for use in a Sustainment Measurement System (SMS), a process designed to evaluate likelihood toward successful sustainment of SAMHSA-funded programs and initiatives and to provide feedback to grantees that would enhance the likelihood of successful sustainment. The SMS combined existing sources of information obtained from SAMHSA grantees described above with information gathered by an instrument that assessed determinants as well as outcomes of sustainment to identify and support both the unique requirements for improving sustainment for individual funding initiatives as well as for developing a generalizable framework of sustainment across diverse prevention approaches, thereby bringing precision to monitoring the structures and processes for sustaining each prevention approach and improving the likelihood of achieving sustainment of any grantee’s prevention efforts, regardless of source of funding [39].

The objective of the current study was to explore and identify dimensions of the Sustainment Measurement System Scale (SMSS) that was designed to assess the sustainment of prevention programs and initiatives by generating quantitative items from qualitative content domains of sustainment outcomes and sustainability determinants and subjecting them to confirmatory factor analysis in order to discern their factor structure. Our goal was to develop a scale that focused on the determinants and outcomes of sustainment of prevention programs and initiatives and to examine its factor structure, reliability, and construct validity.

## Methods

### Background

Representatives from grantees supported by the following seven SAMHSA programs took part in the study: (1) the Sober Truth on Preventing Underage Drinking Act (STOP-Act) grants committed to prevent and reduce alcohol use among youth and young adults; (2) the Implementing Evidence-based Prevention Practices in Schools (PPS) grants designed to address the prevention of early childhood behavioral disorders; (3) the Strategic Prevention Framework State Incentive (SPF-SIG) grants to prevent the onset of substance use amongst youth; (4) the Garrett Lee Smith (GLS) State and Tribal Youth Suicide Prevention grants to address depression, suicidal attempts, and behavioral health problems linked to suicide; (5) the Substance Abuse and HIV Prevention Navigator Program for Racial/Ethnic Minorities (Prevention Navigator, PN) grants serving minority populations at-risk for substance use and HIV; (6) Minority Serving Institutions Partnerships with Community-Based Organizations (MSI-CBOs) grants serving communities at risk for substance use, HIV, and hepatitis-C infections; and (7) the Capacity Building Initiative for Substance Abuse (SA) and HIV Prevention Services for At-Risk Racial/Ethnic Minority Youth and Young Adults (HIV-CBI) grants to prevent and reduce the onset of SA and transmission of HIV/AIDS among at-risk populations ages 13–24.

### Participants

Program Officers representing the 7 SAMHSA funding initiatives provided names and email addresses of all project directors and key personal for each organization currently or previously funded under these initiatives. Researchers identified other people to contact from information provided by project directors of all PPS grantees funded by RFA SM-10-017. Names and email addresses for two to three contacts (i.e., director, coordinator, local evaluator) per GLS grantee site were obtained from ICF international, the GLS contracted evaluators of the grant program, for grantees belonging to Cohorts 8 through 12, per recommendation of SAMHSA program officers. Invitations to participate in the study were sent to 528 representatives of 306 grantee sites supported by the 7 SAMHSA grants between November 2017 and March 2019. One-hundred eighty-six representatives of 145 grantees agreed to participate, resulting in an individual representative response rate of 35.2% and a grantee response rate of 47.5%.

### Procedure

The study was approved by the appropriate Institutional Review Boards prior to participant recruitment, and informed consent was obtained prior to administering surveys. Study data were collected and managed using REDCap electronic data capture tools hosted at Northwestern University. REDCap (Research Electronic Data Capture) is a secure, web-based application designed to support data capture for research studies, providing (1) an intuitive interface for validated data entry, (2) audit trails for tracking data manipulation and export procedures, (3) automated export procedures for seamless data downloads to common statistical packages, and (4) procedures for importing data from external sources [40].

Each participant was emailed an invitation to participate including a link to the web-based survey. Participants reviewed informed consent and after agreeing to participate were able to access the survey and proceed to the survey items. Once participants logged in to the online survey, they were able to answer questions and could pause and resume at any time. The online survey took approximately 15 to 20 min to complete.

### Measures

#### The Sustainment Measurement System Scale (SMSS)

Item development for the SMSS is described in detail elsewhere [41]. Briefly, interviews were conducted with 45 representatives of 10 SAMHSA grantees and 9 SAMHSA government program officers that was comprised of three parts: (1) a series of semi-structured questions relating to experience with implementing and sustaining the grantee’s program; (2) a free list exercise [42] asking participants to respond to whatever came to mind when asked what was meant by the term sustainment or sustainability, what components of their programs they most wanted to sustain, and what it would take to sustain those components; and (3) a template of Consolidated Framework for Implementation Research (CFIR) domains and components [43] in which participants were asked to rate each of the domains and elements as being unimportant (0), somewhat important (1), important (2), or very important (3) to sustainability of their program and to explain the basis for their assessment of each component to sustainability. The CFIR template was used to determine whether the components believed to be associated with successful implementation are also associated with successful sustainment. Templates of the PSAT and SIC sustainability measures informed the semi-structured interview questions and analysis of the free-list exercise.

The data sets containing sustainability elements from each of the three components of the interview (semi-structured interview, free lists, and CFIR checklist) were then compared through a process of data triangulation (i.e., determining consistency of findings obtained from different sources of data) to identify items that were elicited from more than one data set. Items were then placed into three groups: (1) those that appeared in only one of the three data sets, (2) those that appeared on two of the three data sets, and (3) those that appeared on all three data sets. Four sustainability elements were identified by all three data sets: (1) ongoing coalitions, collaborations, networks, and partnerships; (2) infrastructure and capacity to support sustainability; (3) community need for program; and (4) ongoing evaluation of performance and outcomes). An additional 11 elements were identified by two of three data sets: (1) availability of funding; (2) consistency with organizational culture; (3) evidence of positive outcomes; (4) development of a plan for implementation and sustainment; (5) presence of a champion; (6) institutionalization and integration of program; (7) institutional support and commitment; (8) community buy-in and support; (9) program continuity; (10) supportive leadership; and (11) opportunities for staff training [41].

To construct this survey, the fifteen elements were condensed to create a 42-item scale divided into the following subscales:

- *Sustainment outcomes:* 4 items assessing the continued operation of the program or initiative, including delivering prevention services to intended population that are evidence-based as described in the original application for funding and periodically measuring service fidelity.
- *Financial stability:* 6 items measuring funding from federal, state, or local governments and non-profit and non-governmental sources, combination of earmarked and discretionary funding, sustained funding, financial support from diverse community interests, and financial solvency.
- *Responsiveness to community needs and values*: 7 items measuring meeting needs of intended target populations and behavioral health needs of communities/populations being served, adaptability to meet these needs, consistency with norms and values of participating organizations, fit with values of sustaining organizations and communities, shared perception of project importance by participating organizations, and unacceptability of public health problem *addressed by project.*
- *Coalitions, partnerships, and networks*: 8 items measuring networking of grantee organization with other organizations committed to program sustainability, community engagement in development of project goals, community access to knowledge and information about the project, project support by a coalition/partnership/network of community organizatons, network expansion, commitment to continued operation of project, level of networking, and communications within organizations responsible for sustaining the project.
- *Infrastructure and capacity to support sustainment*: 9 items measuring available resources for project implementation and sustainment, integration into operations of the organization and partners, advanced development of plans for implementing and sustaining the project, execution of the project according to these plans, adequacy of staff to sustain program goals and activities, sufficiency of training available to staff and community members, staff knowledge and supportive beliefs, and staff self-efficacy to implement the project.
- *Implementation leadership:* 5 items measuring active engagement of leaders in project implementation and sustainment, involvement of community leaders in the project, appointment of someone responsible for coordinating project implementation and sustainment, support from a program champion, and process in place for sustainment in the event the champion leaves.
- *Monitoring, evaluation, and program outcomes:* 3 items measuring ongoing evaluation of progress made toward sustainment, sufficiency, and timeliness of feedback about project delivery and quality improvement, and evidence of positive outcomes.

Study participants were asked to indicate level of agreement with a series of statements using a Likert scale ranging from 1 (not at all) to 5 (all the time) for the four items contained in one subscale measuring sustainment outcomes and seven different domains of determinants of sustainment; lower scores on all subscales indicate lower levels of agreement, while higher scores indicate higher levels of agreement with the respective statements. Each subscale score is represented as an average of the scores for each item included in the subscale. The average of the scores for each item included in the Sustainment Outcomes subscale was defined as Global Sustainment.

#### Program characteristics

Two sets of variables measuring characteristics of the funded programs and initiatives were included in the analysis to determine whether the association between sustainability determinants and sustainment outcome varied by one or more program characteristics and to assess the convergent and discriminant validity of the SMSS. The first set included a categorical variable describing program type (community or state) and a categorical variable describing program focus (mental health or substance use). The second set also included two variables, one describing whether the grantee is currently funded by one of the seven SAMHSA grant initiatives (yes or no), and if not, a variable describing the current status of the grantee’s program (the project no longer exists, the project continues to exist and it has been substantially adapted, and the project continues to exist in much the same form as it did when funded by the SAMHSA program).

#### Statistical analyses

To evaluate the psychometric properties of the SMSS, confirmatory factor analyses (CFA) with maximum likelihood estimation were conducted using EQS statistical software [44, 45]. The aim of the CFA was to determine if the data fit the hypothesized model of sustainability determinants and sustainment outcomes based on the qualitative research conducted in earlier study described above [41, 45]. CFA is commonly used to confirm a hypothesized model based on theory or prior empirical research, as well as evaluate the reliability and validity of measures employed in implementation research [15, 17, 18, 46–48]. Initially, confirmatory factor analysis was applied to all 42 items to identify poorly performing items and test our hypothesized sustainment domain structure, followed by subsequent modifications based on resulting model modification indices and theoretical justification [49]. Poor items were those that had low reliability or poor fit (i.e., factor loadings below 0.300) with the intended latent factor (or subscale). Principal axis factoring (PAF) with Promax oblique rotation was also conducted to provide guidance on item reduction and construct reclassification. Principal axis factoring was selected for factor extraction because it allows for consideration of both systematic and random error [50], and Promax oblique rotation was utilized as we assumed that the derived factors would be correlated [51]. Three criteria were used to determine the number of factors to retain: (1) examination of the oblique rotated factor pattern matrix, (2) parallel analysis [52], and (3) interpretability of the factor structure as indicated in the rotated solution. Examination of the rotated factor structure included identification of eigenvalues above 1.0 and Scree test results, as well as absence of multicollinearity and presence of outliers [53]. We then used four measures of model fit to assess model adequacy for the initial model and revised models: chi-square statistic, the comparative fit index (CFI), root mean square error of approximation (RMSEA), and the standardized root mean square residual (SRMR). CFI values greater than 0.95, RMSEA values less than 0.06, and SRMR values less than 0.08 indicate model fit that is deemed acceptable [49].

Reliability of the SMSS was assessed by examining Cronbach’s alpha internal consistency for each of the subscales and the total scale. Cronbach’s alpha of .70 and above is considered good internal consistency for a newly developed scale [54]. Corrected item total correlation tests were also conducted in order to check each item’s contribution to the total scale. An item to total correlation higher than .4 was considered acceptable [55]. Item analyses were also conducted, including an examination of inter-item correlations and alpha if the item is removed.

Construct validity of an instrument is typically assessed by comparing constructs or measurements that theoretically should be related to one another to determine if that is the case (convergent validity) and by comparing concepts or measurements that are not supposed to be related to determine if they are actually unrelated (discriminant validity) [56]. In this study, convergent and discriminant validity were assessed by computing Pearson product-moment correlations of SMSS determinant subscales and global sustainment outcome scores, and one-way analysis of variance of mean outcome and determinant subscale scores by current funding from the original SAMHSA initiative (yes/no) and the program status (no longer in operation, exists but has been extensively adapted, exists pretty much in the same form), respectively. Only 5 programs were no longer in operation at the time the study was conducted and therefore removed from the comparison, even though their mean values were lower than those for the other two types of programs. Current funding by the original SAMHSA initiative was chosen to assess the convergent validity of the SMSS as it was hypothesized that grantees currently funded would have higher mean scores of sustainment outcomes than grantees no longer funded. Program status was chosen to assess the divergent validity of the SMSS because the determinants were hypothesized to be unrelated to whether the sustained program was adapted or not as long as it continued to exist.

## Results

**Fig. 1:**
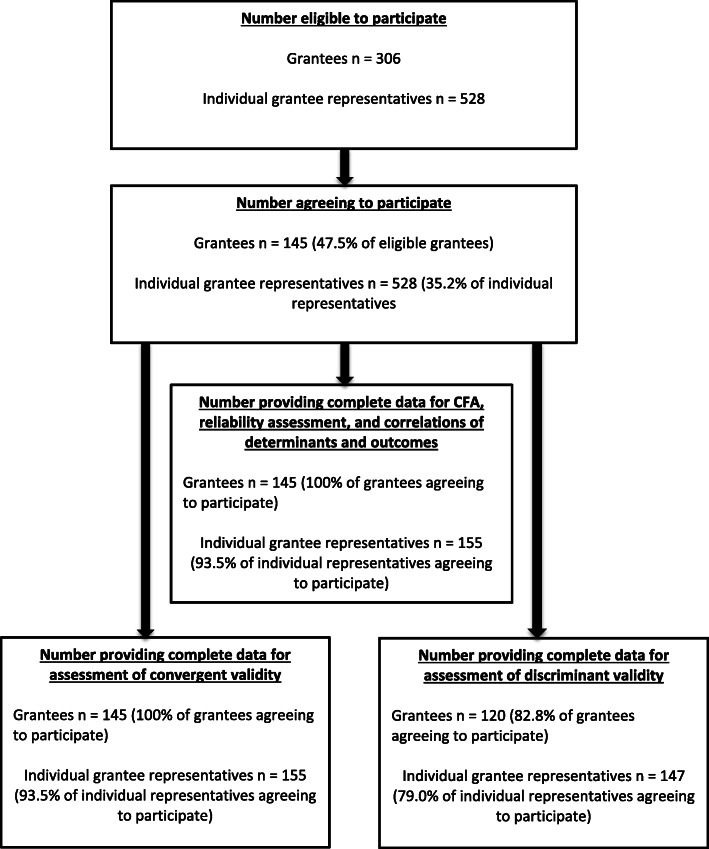
Flowchart of participants at each stage of study

There were no missing values for any of the 4 sustainment outcome variables; however, missing data for each of the determinant variables ranged from 15.8 to 17.9%. The same 29 to 31 individual grantee representatives did not provide responses to any of the determinant items and were removed from analyses of sustainability determinants by sustainment outcomes or determinants by current funding status or program status. As a result, 113 (77.9%) of grantees were represented by one individual, 26 (17.9%) grantees were represented by two individuals, and 6 grantees were represented by 3 (4.1%) individuals. A flow chart describing the numbers of participants from recruitment to data collection to data analysis is presented in Fig. 1. The effects of nesting of individuals within grantees was confirmed by the absence of any differences in associations between determinants and outcomes by grantee and associations between determinants and outcomes by individual participants.

Details of participating representatives and programs used in the data analysis are provided in Table 1. Thirty-four of the grantees (23.4%) and 79 of the grantee representatives (42.5%) focused their efforts on mental health, while 111 (76.6%) grantees and 107 grantee representatives (57.5%) focused on substance use. Furthermore, 92 grantees (63.4%) and 107 grantee representatives (57.5%) addressed these issues at the community level, and 53 grantees (36.6%) and 79 grantee representatives (42.4%) addressed these issues at the state level. Individual representatives included 43 (23.1%) men and 143 (76.9%) women; racial/ethnic groups represented included 138 (74.2%) non-Hispanic whites, 21 (11.3%) African-Americans, 18 (9.7%) Latinx, 5 (2.7%) Asian/Pacific Islanders, and 4 (2.2%) Native Americans; and project roles represented included 15 (7.0%), principal investigators, 89 (47.9%) project directors, 54 (29.4%) coordinators, 19 (10.4%) evaluators, and 8 (4.3%) coalition members.

**Table 1:** Characteristics of participating programs in tests of the sustainment measurement system

	Program type	Program focus	No. of programs	No. of participants
STOP-Act	Community	Substance use	52	60
Positive Prevention in Schools	Community	Mental health	9	10
Strategic Prevention Framework	State	Substance use	28	32
Garrett Lee Smith	State	Mental health	25	47
Prevention Navigator	Community	Substance use	6	7
MSI-CBOs	Community	Substance use	9	10
HIV CBI	Community	Substance use	16	20
Total			145	186

### Confirmatory factor analysis

Results of the CFA analyses are presented in Table 2. In the initial model, we gave no consideration of program type or program focus affecting either the measurement model (e.g., loadings and unique variances) or the structural model (e.g., means, variances, and covariances of the latent variables). Although the 6 sustainment subscales and 1 sustainment outcome subscale exhibited good to excellent reliability, CFA analyses indicated poor fit of the 42-item nine-factor model (labeled Initial Model) across the four major indices (chi-square, CFI, RMSEA, SRMR). Elimination of items with a factor correlation of less than .300 resulted in 36 items. Results of an exploratory factor analysis revealed 10 domains that included a segmentation of the responsiveness to community needs and values subscale into two subscales—responsiveness to community needs (2 items) and responsiveness to community values (3 items)—and a segmentation of infrastructure and capacity subscale into two subscales—organizational capacity (4 items) and organizational staff capability (3 items). Only 1 item (community leadership) loaded onto the 10th factor, which was also eliminated for further analysis. One of the items from the leadership subscale also exhibited a much higher loading in the coalition, partnerships, and networks factor (.660) than on the leadership factor (.129). The resulting 35-item nine factor model (labeled Final Model) provided better fit with an absolute model fit of *χ*^2^(517) = 960.23, *p* < .001, an RMSEA of .076 and an SRMR of .066 meeting criteria for acceptability; however, fit was not supported by a CFI of .95 or greater. The standardized factor loadings ranged from .56 to .88, and all were statistically significant (all *p* levels < 0.001). Separating the responsiveness to community needs and values subscale into two separate subscales also increased the Cronbach’s alpha coefficient from .60 to .70 for both subscales. The 35-item version of the SMSS is provided in the Appendix.

**Table 2:** Item-factor loadings for initial and final itemized subscales of the sustainment measurement system scale

Subscale definition and items	Initial model		Final model			
	Item factor loading	*R*^2^	Item factor loading	*R*^2^	Mean	S.D.
Inter-item reliability of entire scale	.93		.93			
Factor 1. Financial stability	*α* = .75		*α* = .81		2.58	1.04
The project is supported by federal, state, or local government funding.	.090	.008				
Project is funded through non-profit, private, and/or non-governmental sources.	.579	.335	.582	.339	1.94	1.32
Project has a combination of stable (i.e., earmarked) and flexible (i.e., discretionary) funding.	.745	.555	.748	.559	2.40	1.34
Project has sustained funding.	.758	.575	.754	.569	2.69	1.37
Diverse community organizations are financially invested in the success of the project.	.720	.519	.724	.524	2.77	1.45
Project is financially solvent.	.585	.342	.581	.338	3.15	1.44
Factor 2. Responsiveness to community needs and values	*α* = .60					
Factor 2a. Responsiveness to community needs			*α* = .70		4.47	0.64
Project meets the needs of the intended target populations.	.548	.300	.871	.758	4.56	0.68
Project addresses the behavioral health needs of the communities/populations being served.	.460	.212	.557	.310	4.38	0.78
Factor 2b. Responsiveness to community values			*α* = .70		4.72	0.42
Project can be adapted to meet the needs of the communities or populations being served.	.489	.239	.437	.191	4.61	0.60
Project is consistent with the norms, values, and guiding principles of participating organizations.	.683	.467	.761	.580	4.81	0.44
Project fits well with the values of the organization(s) responsible for sustaining it and the communities where it is being sustained.	.756	.572	.875	.766	4.73	0.54
Participating organizations have a shared perception of the importance of the project.	.382	.146				
The current social or health issue addressed by the project is perceived as intolerable or unacceptable to the community.	.144	.021				
Factor 3. Coalitions, partnerships, and networks	α = .92		α = .93		4.11	0.82
Grantee organization is networked with other organizations committed to sustaining the project.	.504	.254				
Community members are passionately committed to sustaining the project.	.675	.455	.691	.477	4.09	0.92
Community is actively engaged in the development of project goals.	.699	.489	.723	.522	3.87	1.03
Community has access to knowledge and information about the project.	.688	.473	.710	.504	4.20	0.93
Project is supported by a coalition/partnership/network of community organizations.	.825	.681	.827	.684	4.32	1.00
Coalition/partnership/network members actively seek to expand the network of community organizations, leaders, and sources of support for this project.	.862	.744	.855	.732	4.05	1.08
Coalition/partnership/network is committed to the continued operation of this project.	.880	.774	.858	.736	4.18	1.05
High level of networking and communication within the organizations responsible for sustaining the project.	.835	.697	.829	.687	4.10	1.04
Community leaders are actively involved in the project.			.750	.562	3.97	0.98
Factor 4. Infrastructure, capacity and support	*α* = .88					
Available resources dedicated for implementing and sustaining the project.	.549	.301				
Project has adequate staff to sustain the program’s goals and activities.	.630	.397				
Factor 4a. Organizational capacity			*α* = .85		3.76	0.95
Project exhibits sound fiscal management.	.657	.432	.645	.416	4.52	0.85
Project is well integrated into the operations of the organization and its partners.	.794	.631	.806	.650	4.25	0.86
Plans for implementing and sustaining the project are developed in advance.	.684	.468	.770	.593	3.95	0.96
Project is carried out or accomplished according to those plans.	.748	.560	.829	.688	4.19	0.92
Factor 4b. Organizational staff capability			*α* = .82		4.56	0.68
Project offers sufficient training to agency staff and community members.	.635	.404	.747	.558	4.34	0.93
Staff possesses adequate knowledge and supportive beliefs about the project.	.704	.496	.884	.781	4.70	0.71
Staff feel themselves to be capable of implementing the project.	.646	.417	.766	.587	4.63	0.71
Factor 5. Implementation leadership	*α* = .79		*α* = .74		4.06	0.83
Leaders in the organization or coalition/partnership/network are actively engaged in the process of implementing and sustaining the project.	.671	.451				
Community leaders are actively involved in the project.	.729	.532				
The project has a formally appointed person responsible for coordinating the process of implementing and sustaining the project.	.661	.437	.662	.439	4.52	4.09
The project is also supported by a champion who is actively engaged in the process of implementing and sustaining the project.	.660	.435	.783	.613	4.09	0.96
We have a process in place to sustain the project in the event our champion leaves.	.623	.388	.711	.506	3.56	1.15
Factor 6. Evaluation, feedback, and program outcomes	*α* = .74		*α* = .75		4.08	0.78
Ongoing evaluation of progress made towards sustainment.	.654	.428	.656	.431	3.95	1.06
Sufficient and timely feedback about the project delivery to maintain or improve quality.	.843	.711	.832	.692	4.11	1.00
Evidence of positive outcomes	.559	.312	.568	.322	4.17	0.79
Factor 7. Sustainment outcomes	*α* = .84		*α* = .85		4.41	0.74
Continue to operate as described in original application	.608	.370	.608	.370	4.36	0.82
Continue to deliver preventive services to intended population	.828	.685	.834	.695	4.67	0.72
Continue to deliver evidence-based services	.741	.548	.743	.553	4.54	0.84
Periodically measure fidelity of services delivered	.745	.554	.734	.539	4.09	1.18
Total subscales	7		9			
Total items	42		35			
*X*^2^ (d.f.)	1577.1 (798)		960.2 (524)			
CFI	0.756		0.844			
RMSEA	0.082		0.076			
SRMR	0.090		0.066			

Table 3 shows the correlations among the SMSS subscales. In general, the correlations between the responsiveness to community values subscale and the other eight subscales (average *r* = .19) were lower than the correlations among the other eight subscales (average *r* = .42). Of particular note, sustainment outcome was significantly associated with all 8 determinants (average *r* = .43), with significance levels < 0.001 except for financial stability (*p* < 0.01).

**Table 3:** Factor correlations of global sustainment outcomes and sustainment determinants

		1	2	3	4	5	6	7	8	9
1	Global sustainment outcomes	–								
2	Financial stability	.22**	–							
3	Responsiveness to community needs	.51***	.20*	–						
4	Responsiveness to community values	.35***	.09	.35***	–					
5	Coalitions, partnerships, & networks	.53***	.47***	.35***	.05	–				
6	Organizational capacity	.38***	.40***	.32***	.17*	.50***	–			
7	Organizational staff capability	.59***	.18*	.38***	.23**	.53***	.47***	–		
8	Implementation leadership	.38***	.35***	.20*	.16	.57***	.58***	.43***	–	
9	Evaluation, feedback, & program outcome	.49***	.35***	.41***	.14	.53***	.45***	.53***	.54***	–

*p < 0.05, **p < 0.01, ***p < 0.001

### Convergent and discriminant validity

Table 4 shows the results of correlations between the global sustainment outcome score and the 8 determinant subscale scores stratified by program type and focus. Global sustainment was significantly associated with all 8 determinants in community-based programs and programs targeting substance use prevention. Global sustainment was associated with responsiveness to community needs and values and organizational capacity and staff capability in statewide prevention programs, and with six of the eight determinants (all but financial stability and responsiveness to community needs) in programs that focused on mental health.

**Table 4:** Correlations of global sustainment outcomes by sustainment determinants, program type, and program focus

Determinant	Program type		Program focus	
	Community (*n* = 86)	State (*n* = 69)	Substance use (*n* = 104)	Mental health (*n* = 51)
Financial stability	.33**	.04	.21*	.24
Responsiveness to community needs	.54***	.43***	.69***	.12
Responsiveness to community values	.25*	.58***	.38***	.31*
Coalitions, partnerships and networks	.67***	.17	.57***	.44***
Organizational capacity	.58***	.36**	.52***	.42**
Organizational staff capability	.60***	.56***	.58***	.63***
Implementation leadership	.48***	.19	.41***	.32*
Evaluation, feedback, & program outcomes	.64***	.20	.47***	.51**

*p < 0.05, **p < 0.01, ***p < 0.001

A comparison of sustainment determinants and outcomes by program current funding status in provided in Table 5. Participants of programs no longer funded by the original SAMHSA initiative reported significantly less global sustainment (*F* = 37.67, d.f. = 154, *p* < 0.001) as well as the four individual forms of sustainment. They also reported significantly less responsiveness to community needs (*F* = 5.14, d.f. = 127, *p* = 0.025), coalitions, partnerships, and networks (*F* = 6.99, d.f. = 125, *p* = 0.009), organizational staff capability (*F* = 7.34, d.f. = 125, *p* = 0.008), and evaluation, feedback, and outcomes (*F* = 9.37, d.f. = 117, *p* = 0.003).

**Table 5:** Mean individual and global sustainment outcomes and sustainment determinants by current program funding

Construct	Currently funded by SAMHSA grant initiative	
	Yes	No
Outcome	(*n* = 137)	(*n* = 18)
Continue to operate as described in original application	4.45 (0.72)	3.56 (1.10)***
Continue to deliver preventive services to intended population	4.80 (0.56)	3.83 (1.10)***
Continue to deliver evidence-based services	4.65 (0.71)	3.78 (1.26)***
Periodically measure fidelity of services delivered	4.23 (1.03)	2.94 (1.55)***
Global sustainment	4.53 (0.56)	3.53 (1.14)***
Determinant	(*n* = 113)	(*n* = 15)
Financial stability	2.70 (0.99)	2.67 (1.03)
Responsiveness to community needs	4.53 (0.56)	4.13 (1.08)*
Responsiveness to community values	4.73 (0.39)	4.76 (0.53)
Coalitions, partnerships, and networks	4.19 (0.70)	3.60 (1.33)**
Organizational capacity	4.32 (0.64)	4.16 (0.86)
Organizational staff capability	4.62 (0.53)	4.14 (1.22)**
Implementation leadership	4.12 (0.76)	3.89 (1.00)
Evaluation, feedback, & program outcomes	4.11 (0.69)	3.42 (1.11)**

*p < 0.05, **p < 0.01, ***p < 0.001

The discriminant validity of the SMSS was assessed in comparisons of total sustainment outcomes and determinants by program status. The results are presented in Table 6 below. The global sustainment outcome score and the individual measures of sustainment were significantly greater in programs that continued to exist in the same form. However, only one determinant was found to be significantly greater in such programs (organizational staff capability; *F* = 4.52, d.f. = 146, *p* = 0.035), suggesting strong support for the discriminant validity of the SMSS.

**Table 6:** Mean individual and global sustainment outcomes and sustainment determinants by current program status

Construct	Continues to exist but adapted	Continues to exist in same form
Outcome	(*n* = 55)	(*n* = 125)
Continue to operate as described in original application	4.00 (0.94)	4.53 (0.70)***
Continue to deliver preventive services to intended population	4.40 (0.95)	4.81 (0.52)***
Continue to deliver evidence-based services	4.24 (1.09)	4.69 (0.65)***
Periodically measure fidelity of services delivered	3.71 (1.49)	4.29 (0.97)**
Global sustainment	4.09 (0.99)	4.58 (0.51)***
Determinant	(*n* = 51)	(*n* = 96)
Financial stability	2.80 (1.17)	2.49 (0.96)
Responsiveness to community needs	4.48 (0.76)	4.48 (0.57)
Responsiveness to community values	4.72 (0.45)	4.73 (0.38)
Coalitions, partnerships, and networks	4.05 (1.05)	4.13 (0.67)
Organizational capacity	4.19 (0.84)	4.26 (0.68)
Organizational staff capability	4.41 (0.87)	4.65 (0.50)*
Implementation leadership	4.16 (0.98)	4.00 (0.74)
Evaluation, feedback, & program outcomes	4.01 (0.91)	4.10 (0.65)

*p < 0.05, **p < 0.01, ***p < 0.001

## Discussion

Although there exist other measures designed to evaluate sustainability of evidence-based programs and interventions from the point of view of the determinants of sustainability or sustainment as an outcome, the Sustainment Measurement System Scale (SMSS) is the first instrument designed to assess both sustainability determinants and sustainment outcomes. Further, the SMSS is designed to assess different prevention programs of different types and foci. As such, it contains elements that are specific to particular programs but enable comparisons across different types of programs. It also demonstrates some potential as a tool for providing feedback to organizations, enabling them to monitor their trajectory towards achieving the final stage of state of implementation and increase the likelihood of successfully doing so.

The SMSS draws from and shares several features of existing measures of sustainability. For instance, like the Stages of Implementation Completion Scale [12], the SMSS measures sustainment as an outcome and can be adapted for use as a tool for monitoring progress toward sustainment [13]. Unlike the SIC, it measures characteristics of programs and their inner and outer settings that predict these outcomes and excludes earlier phases of implementation. Like the Program Sustainability Assessment Tool [15], Sustained Implementation Support Scale [17], and Program Sustainability Index [18], the SMSS measures determinants of sustainability and exhibits similar internal consistency, model fit, and construct validity. With the possible exception of responsiveness to community values, the domains are highly correlated with one another. SMSS has similar chi-square significance, RMSS, and SRMR as the PSAT. All of these instruments have less than desirable CFI of .95, suggesting room for improvement.

The SMSS is the product of a standardized process of eliciting determinants and outcomes of sustainability that are both specific to the program being sustained and generalizable to other types of programs and initiatives [41]. This approach is also more consistent with the Dynamic Sustainability Framework [5] and the growing consensus of sustainability as a dynamic process with changes in both determinants and outcomes over time. In this study, correlations between sustainability determinants and sustainment outcomes were stronger with community-based programs and programs targeting substance use than with state-based programs and programs targeting mental health. However, there was consistency across programs by type and focus with respect to certain determinants such as responsiveness to community values (which included adaptability), organizational capacity, and organizational staff capability.

The design and evaluation of the SMSS also provides some important insights into sustainment as the final stage of the implementation of evidence-based prevention programs and initiatives. The process of achieving sustainment is the product of eight domains of sustainability determinants: financial stability; responsiveness to community needs; responsiveness to community values; coalitions, partnerships, and networks; organizational capacity; organizational staff capability; implementation leadership; and evaluation and feedback and positive program outcomes. Luke and colleagues [15] found similar associations between program manager and staff perceptions of sustainability of their programs and the determinants of funding stability, partnerships, organizational capacity, and program evaluation. Hodge and colleagues [17] found similar associations between sustained implementation of the Triple P parent training program and the determinants of program benefits, workplace support, and leadership style and. Mancini and Marek [18] found similar associations between meeting at risk needs and the determinants of leadership competence, effective collaboration, demonstrating program results strategic funding, staff involvement and integration, and program responsivity. In our study, these associations are consistent across community level programs and/or a focus on substance use prevention, less so with state level programs, and/or a focus on mental health. Many of these determinants decline once funding that supported their implementation comes to an end, which is to be expected since many of them like coalitions, partnerships and networks, staff capability (e.g., training), and the capacity for conducting evaluations and providing feedback are resource-dependent. As predicted, however, it makes no difference whether the program continues to exist in same form as originally proposed or has been adapted to improve fit, also consistent with the Dynamic Sustainability Framework. This may be because many programs are not necessarily implementing a specific practice, and some are just trying to sustain coalitions.

Nevertheless, there are a number of factors that limit the findings of this study. The findings reflect the experience of specific prevention programs and initiatives that have been or are currently funded by SAMHSA; thus, their generalizability to prevention programs funded by other sources (e.g., state or local funds, research grants), to programs targeting treatment, or event to other SAMHSA-funded programs, is limited. The confirmatory factor analysis was constrained by the small number of programs and program representatives. Only two items loaded onto one of the identified constructs (responsiveness to community needs), while only three items loaded onto the construct of responsiveness to community values. Further research is required to determine whether or not they represent two distinct constructs.

With these limitations in mind, future steps include the following: (1) evaluation of the utility of the SMSS with other types of programs in other settings, (2) use of larger samples to confirm fit of the data to the model, (3) development of guidelines for providing feedback to organizations seeking to sustain programs and initiatives based on ongoing monitoring efforts using the SMSS, and (4) consistent with the SMS process, ongoing revision of the tool itself.

## Conclusion

The SMSS is innovative in three specific respects. First, it draws upon the experience of evaluating sustainability in different types of prevention programs and initiatives with different aims and areas of emphases. This enabled us to identify a set of common elements of sustainment that can be used to generate a model and set of testable hypotheses that apply to a broad array of substance use/mental disorder/suicide prevention programs, practices and initiatives, regardless of objectives, outcomes, and infrastructure to achieve these outcomes. Second, although we did not assess the effectiveness of the various programs, practices, and initiatives participating in this study, a measure like the SMSS offers the potential to determine whether the extent to which a program or practice is evidence-based or evidence-informed determines whether it can be sustained. Such information would be invaluable to determining whether the program or practice merits initial or continued funding. Third, this measure can be used to monitor progress toward sustainment and provide feedback to stakeholders as to how to increase the likelihood of sustainment. The SMSS in particular and the SMS in general can be used as a tool for program management as well as research purposes. Although the SMSS was based on programs funded by SAMHSA, the instrument should have general applicability across diverse federal, statewide, and local prevention implementation initiatives.

## Data Availability

The datasets collected and analyzed during the current study will be available from the corresponding author on reasonable request.

## Appendix

### Sustainment Measurement System Scale

INSTRUCTIONS: Please respond to the following statements relating to your [ ]-funded project using a scale ranging from 1 = little or no extent to 5 = a great extent. *If this project no longer exists or no longer provide services, answer based on the experience during the period when [ ]-funded the project. If you feel the statement is not relevant to your program, answer N/A.*

**Table:**

**Sustainment indicators**	**Responses**
1. The project continues to operate as described in the original application for funding.	1 2 3 4 5 N/A
2. The project continues to deliver prevention services to its intended population.	1 2 3 4 5 N/A
3. The project continues to deliver prevention services that are evidence-based.	1 2 3 4 5 N/A
4. This project periodically measures the fidelity of the prevention services that are delivered.	1 2 3 4 5 N/A
**Funding and financial support**	**Responses**
5. The project is funded through non-profit, private, and/or non-governmental sources.	1 2 3 4 5 N/A
6. The project has a combination of stable (i.e., earmarked) and flexible (i.e., discretionary) funding.	1 2 3 4 5 N/A
7. The project has sustained funding.	1 2 3 4 5 N/A
8. Diverse community organizations are financially invested in the success of the project.	1 2 3 4 5 N/A
9. The project is financially solvent	1 2 3 4 5 N/A
**Responsiveness to community needs**	**Responses**
10. The project delivered meets the needs of the intended target populations.	1 2 3 4 5 N/A
11. The project addresses the behavioral health needs of the communities/populations being served.	1 2 3 4 5 N/A
**Responsiveness to community values**	**Responses**
12. The project can be adapted to meet the needs of the communities or populations being served.	1 2 3 4 5 N/A
13. The project is consistent with the norms, values and guiding principles of participating organizations.	1 2 3 4 5 N/A
14. The project fits well with the values of the organization(s) responsible for sustaining it and the communities where it is being sustained.	1 2 3 4 5 N/A
**Coalitions, partnerships, and networks**	**Responses**
15. The community members are passionately committed to sustaining the project.	1 2 3 4 5 N/A
16. The community is actively engaged in the development of project goals.	1 2 3 4 5 N/A
17. The community has access to knowledge and information about the project.	1 2 3 4 5 N/A
18. The project is supported by a coalition/partnership/network of community organizations.	1 2 3 4 5 N/A
19. Coalition/partnership/network members actively seek to expand the network of community organizations, leaders, and sources of support for this project.	1 2 3 4 5 N/A
20. The coalition/partnership/network is committed to the continued operation of this project.	1 2 3 4 5 N/A
21. There is a high level of networking and communication within the organizations responsible for sustaining the project.	1 2 3 4 5 N/A
22. Community leaders are actively involved in the project.	1 2 3 4 5 N/A
**Infrastructure and capacity to support sustainment**	**Responses**
23. The project exhibits sound fiscal management.	1 2 3 4 5 N/A
24. The project is well integrated into the operations of the organization and its partners.	1 2 3 4 5 N/A
25. Plans for implementing and sustaining the project are developed in advance.	1 2 3 4 5 N/A
26. The project is carried out or accomplished according to those plans.	1 2 3 4 5 N/A
27. The project offers sufficient training to agency staff and community members.	1 2 3 4 5 N/A
28. Staff possesses adequate knowledge and supportive beliefs about the project.	1 2 3 4 5 N/A
29. Staff feel themselves to be capable of implementing the project.	1 2 3 4 5 N/A
**Implementation leadership**	**Responses**
30. The project has a formally appointed person responsible for coordinating the process of implementing and sustaining the project.	1 2 3 4 5 N/A
31. The project is also supported by a champion who is actively engaged in the process of implementing and sustaining the project.	1 2 3 4 5 N/A
32. We have a process in place to sustain the project in the event our champion leaves.	1 2 3 4 5 N/A
**Evaluation, feedback, and evidence of positive outcomes**	**Responses**
33. There is ongoing evaluation of progress made towards sustainment.	1 2 3 4 5 N/A
34. There is sufficient and timely feedback about the project delivery to maintain or improve quality.	1 2 3 4 5 N/A
35. The project provides strong evidence of positive outcomes.	1 2 3 4 5 N/A
